# Effectiveness and Safety of Non-Vitamin K Oral Anticoagulants in Non-Valvular Atrial Fibrillation Patients: Results of A Real-World Study in a Metropolitan Area of Northern Italy

**DOI:** 10.3390/jcm10194536

**Published:** 2021-09-30

**Authors:** Emanuele Crocetti, Sarah Cattaneo, Walter Bergamaschi, Stefano De Servi, Antonio Giampiero Russo

**Affiliations:** 1 Epidemiology Unit, Agency for Health Protection (ATS) of Milan, C.so Italia, 19, 20122 Milan, Italy; emanuelecrocetti@yahoo.com; 2 Pharmacy Department, Agency for Health Protection (ATS) of Milan, C.so Italia, 19, 20122 Milan, Italy; scattaneo@ats-milano.it; 3 General Directorate, Agency for Health Protection (ATS) of Milan, C.so Italia, 19, 20122 Milan, Italy; wbergamaschi@ats-milano.it; 4 Department of Molecular Medicine, University of Pavia, 27100 Pavia, Italy; stefano.deservi01@gmail.com

**Keywords:** atrial fibrillation, new oral anticoagulant agents, effectiveness, safety, real-world

## Abstract

Background: New oral anticoagulant agents (NOACs) are valid alternatives for vitamin K antagonists (VKA) in patients with non-valvular atrial fibrillation (NVAF) for stroke prevention. In clinical practice, NOACs users may differ from patients enrolled in clinical trials in age or comorbidities, and thus it is a critical issue to evaluate the effectiveness and safety of NOACs in the real-world. Accordingly, we assessed two-year overall mortality and hospital admissions for myocardial infarction, stroke or bleeding in patients with NVAF users of NOACs compared to warfarin-treated patients. Methods: This is a population-based retrospective new user active comparator study. All atrial fibrillation patients who were naïve and not switcher users of oral anticoagulants from January 2017 to December 2019 were included (*n* = 8543). Data were obtained from the electronic health records of the Milan Agency for Health Protection, Italy. Two-year risks for overall mortality, myocardial infarction, stroke and bleeding were computed using Cox models. Age, sex, number of comorbidities, use of platelet aggregation inhibitors and Proton pump inhibitors and area of residence were used as confounding factors. We also controlled by indication bias-weighting NOACs and warfarin users based on the weights computed by a Kernel propensity score. Results: For all NOACs, we found a decrease in the risks compared with warfarin for mortality (from −25% to −49%), hospitalization for myocardial infarction (from −16% to −27%, statistically significant for apixaban, edoxaban and rivaroxaban) and ischemic stroke (from −23% to −41%, significant for dabigatran and apixaban). The risk of bleeding was decreased for rivaroxaban (−33%) and numerically but not significantly for the other NOACs. Conclusions: After two years of follow-up, in comparison with warfarin, NOACs users showed a significant reduction of overall mortality (all NOACs), hospital admission for myocardial infarction (apixaban and edoxaban), ischemic stroke (dabigatran) and bleeding (rivaroxaban).

## 1. Introduction

New oral anticoagulant agents (NOACs: dabigatran (Pradaxa©: Boehringer Ingelheim am Rhein Germany), rivaroxaban (Xarelto©: Bayer AG Kaiser), apixaban (Eliquis©: Pfizer Manufacturing Freiburg, Germany) and edoxaban (Lixiana©: Daiichi Sankyo Germany)) are all authorized in Europe as valid alternatives for vitamin K antagonists (VKA) in patients with non-valvular atrial fibrillation (NVAF) for stroke prevention [1,2,3,4]. In addition, they have also been approved for treatment and prevention of deep vein thrombosis and pulmonary embolism, as well as for adult patients undergoing elective hip or knee replacement surgery (rivaroxaban, dabigatran and apixaban) [1,2,3,4].

All NOACs have been shown to be at least as effective and safe as warfarin [5]. Furthermore, these drugs have characteristics that make them more convenient to administer than VKA: rapid onset/offset of action, fewer drug interactions and above all, predictable pharmacokinetics which eliminate the need for coagulation monitoring [6].

Therefore, their use has speedily increased [7,8,9]. In Italy, at the end of 2018, almost 1 million citizens, considering only people with NVAF, were treated with NOACs [10].

In clinical practice, NOACs users may differ from patients enrolled in clinical trials in age or comorbidity [11] and in the intensity of the clinical follow-up [12]. Therefore, it is a critical issue to evaluate the effectiveness and safety of NOACs in the real-world since their introduction [7,12,13,14,15,16,17].

However, NOACs have not been placed on the market at the same time, so real-world evidence is lower for those which have entered more recently [18]. In Italy, dabigatran and rivaroxaban entered the market in 2013, apixaban at the beginning of 2014 and edoxaban from September 2016 [10].

Accordingly, in the present manuscript, we sought to assess two-year risk of overall mortality and hospital admissions for myocardial infarction, stroke or bleeding in a large cohort of NVAF patients who were naïve users of each NOAC in the Italian universal healthcare setting, compared to patients treated with warfarin.

## 2. Methods

### 2.1. Study Design

This is a population-based retrospective cohort study conducted on residents in the area of the Milan Health Protection Agency (AHP, about 3.5 million), Italy, who used anticoagulant agents.

In particular, this is a new user active comparator study [19] that includes all new users of warfarin, rivaroxaban, dabigatran, apixaban or edoxaban from 1 January 2017 to the end of December 2019.

### 2.2. Selection and Description of the Cohort

Anticoagulant users were identified among the residents in the AHP area, enrolled in the regional universal coverage healthcare system. Anticoagulant users were identified among the permanent residents in the AHP area registered in the regional universal coverage healthcare system.

We selected citizens who had an anticoagulant prescription in 2017–2019 of warfarin, rivaroxaban, dabigatran, apixaban or edoxaban (*n* = 103,115 patients). We defined new users as those who had no prescriptions for any such drugs in the year prior to the first specific prescription (index date, 48,080 patients).

In addition, we included only patients who did not switch from one anticoagulant to another during the study period (*n* = 43,094). We also considered patients who had a time interval of less than 180 days between two following prescriptions. Therefore, we selected 33,674 NOACs or warfarin users.

Among them, we checked hospital admissions for NVAF. We restricted the time of admission to one year before or one month after the index date, and we considered the following ICD-9 codes for diagnosis: 427.3 Atrial fibrillation and flutter, 427.31 Atrial fibrillation and 427.32 Atrial flutter, and the following ones for procedures: 99.6 Conversion of cardiac rhythm, 99.61 Atrial cardioversion, 99.62 Other electric counter shock of heart, 37.33 Excision or destruction of other lesion or tissue of heart, open approach, and 37.34 Excision or destruction of other lesion or tissue of heart, other approach [20,21]. We excluded patients with mitral stenosis (ICD-9 code 3940) and patients who had cardiac surgery with heart valve replacement (ICD-9 code V433). Overall, we included in the study 8543 people with NVAF, as new users of warfarin or dabigatran, rivaroxaban, apixaban or edoxaban.

Data anonymization was provided by an internal code used in every administrative database and was used for deterministic record linkage to a unique identifier.

This study is ethically compliant with the National Law (D.Lgs. 101/2018 https://www.gazzettaufficiale.it/eli/id/2018/09/04/18G00129/sg, accessed on 30 July 2021) and the “General Authorisation to Process Personal Data for Scientific Research Purposes” (No. 8 and 9/2016, referred to in the Data Protection Authority action of 13 December 2018 https://www.garanteprivacy.it/home/docweb/-/docweb-display/docweb/9068972, accessed on 30 July 2021). Administrative permission to perform the study was granted by the General Direction of the Agency for Health Protection (ATS) of Milan, resolution No. 36 16 January 2018).

### 2.3. Individual and Treatment Characteristics

Demographic variables were extracted from the AHP’s current administrative healthcare databases.

Anticoagulant treatment was determined using the drug prescription database and selecting the following codes of the Anatomical Therapeutic Chemical (ATC) classification system (https://www.whocc.no, accessed on 30 July 2021): B01AA03 (warfarin), B01AE07 (dabigatran), B01AF01 (rivaroxaban), B01AF02 (apixaban) and B01AF03 (edoxaban).

Comorbidities were identified by algorithms that identify patients with chronic diseases in the Lombardy region [22,23]. Comorbidities include diagnosis of cancer history, diabetes, hypertension, congestive heart failure, peripheral artery disease, chronic obstructive pulmonary disease, chronic kidney failure, neurologic diseases (including epilepsy, Parkinson’s disease, Alzheimer, multiple sclerosis, neuromyelitis optica and dementia), autoimmune diseases (including rheumatoid arthritis, lupus erythematosus, systemic sclerosis, Sjogren’s diseases, ankylosing spondylitis, myasthenia gravis, Hashimoto’s thyroiditis and autoimmune hemolytic anemia) and endocrinopathies (including acromegaly and gigantism, diabetes insipidus, Addison’s disease, hyper- and hypo-parathyroidism, congenital and acquired hypothyroidism). We used the number of comorbidities as a general health status index for propensity score and adjustment, splitting patients into two classes (≤4, 4+).

We also selected for each patient some other therapies on the basis of the prescriptions received during the same year of the index date. In particular, aiming to identify chronic therapies, we selected drugs for which there were at least three prescriptions in the year. Specifically, we considered the following prescribed drugs based on the first 5-digit codes of the ATC classification: B01AC (platelet aggregation inhibitors excluding heparin (PAI)) and A02BC (proton pump inhibitors (PPI)).

We also considered six sub-areas of residence (ASST) within the AHP: Milan (the main city), Rhodese, North Milan, West Milan, Melegnano and Lodi, in order to take into account drug prescription attitudes of the primary care groups of general practitioners and the interaction with the hospital and healthcare facilities of the territory.

### 2.4. Definition of the Outcome

NVAF patients were followed up until 31 December 2019, for the status of life. In addition, we checked the admissions database for the first admission after the index date and before 1 January 2020, for myocardial infarction, stroke or bleeding. Myocardial infarction and stroke were defined with the algorithms established by the Lombardy region [22,23]. Bleeding was defined as any hemorrhagic event leading to hospitalization using the following ICD9-CM codes in hospitalizations occurring during the years 2017–2019: 285.1, 362.81, 363.8, 364.41, 372.72, 374.81, 376.32, 379.23, 388.69, 423.0, 430, 431, 432*, 456.0–456.2, 459.0, 530.21, 530.21, 530.7, 530.82, 531.0, 531.2, 531.4, 531.6, 532.0, 530.2, 530.4, 530.6, 533.0, 530.2, 530.4, 530.6, 534.0, 530.2, 530.4, 530.6, 535.01, 537.83, 557.0, 562.02, 562.03, 562.12, 562.13, 569.3, 569.85, 569.86, 578.0–578.9, 578.1, 578*, 595.0, 599.7, 626.2, 626.6, 784.4, 784.7, 786.3, 852* and 853*.

People were followed from the index date to the date of each of all possible events or until the end of follow-up (31 December 2019). To reduce the possible bias due to the different time of availability on the market of NOACs in the analysis, the maximum follow-up was truncated to 2 years.

### 2.5. Statistical Analysis

For each NOAC, the characteristics of the analyzed population were presented as frequencies or means, and compared with warfarin by *t*-test, or chi-squared test when appropriate.

To adjust for potential confounding due to indication bias, for each comparison between any different type of NOAC and warfarin, we applied a propensity score Kernel weighting. The propensity score approach has been applied to make the sample distribution of the observed covariates very similar in treated (any single NOAC) and untreated (warfarin) groups, assuring that any difference is mainly attributed to the treatment itself and that the covariates (sex, age, number of comorbidities, use of PAI, use of PPI and sub-area of residence) and treatment become unrelated in both groups. This technique may have the potential to estimate the casual effect of the treatment [24]. The applied Kernel estimation uses all available cases and matches treatment units to a weighted mean of all control units.

Table 1 includes Rubin’s B and R statistics. Rubin’s B is the absolute standardized difference of the means of the linear index of the propensity score in the treated and non-treated groups [25].

Rubin recommends that B should be less than 25 and that R be between 0.5 and 2 for the samples to be considered sufficiently balanced [25].

For each NOAC, multivariate hazard ratios (HR) and 95% confidence intervals (CIs) for overall mortality, hospitalization for myocardial infarction, stroke or bleeding were calculated using Cox models, in comparison with warfarin. All the variables used for propensity score computation were included in each multivariate model. Moreover, we also included the Kernel weights as the weighting variable in the full models. A two-tailed *p*-value of less than 0.05 was considered significant for all tests. All statistical analyses were conducted using Stata Statistical Software: Release 12. College Station, TX: StataCorp LP.

## 3. Results

The characteristics of the 8543 NVAF patients included in the analysis are shown in Table 2: 12.9% had prescriptions for warfarin, 19.2% for dabigatran, 16.2% for rivaroxaban, 31.5% for apixaban and 20.2% for edoxaban.

The mean age at the start of treatment was lower for patients prescribed rivaroxaban than warfarin (73.9 years vs. 75.4, *p* < 0.001), and higher for both edoxaban (78.7, *p* < 0.001) and apixaban (79.0, *p* < 0.001). The proportion of women was higher among patients treated with apixaban (52.5%, *p* < 0.001) and edoxaban (54.6%, *p* < 0.001) than patients prescribed warfarin (44.2%).

Patients using NOACs had a significantly lower average number of comorbidities (from 2.0 to 2.1) than those using warfarin (2.3, *p* < 0.01). The proportion of patients using platelet inhibitors did not differ among groups (mean 4.2%). The use of PPI was lower for all the NOACs users (from 55.0% to 62.5%) than for warfarin users (70.7%, *p* < 0.01).

Concerning residence area, the proportion of those residing in Milan was greater for those prescribed rivaroxaban (39.6%, *p* < 0.05), edoxaban (40.4%, *p* < 0.05) and apixaban (48.0, *p* < 0.01) than for warfarin users (35.5%).

The mean follow-up (for mortality) was longer for dabigatran, apixaban (2.2 years, *p* < 0.05) and rivaroxaban (1.27, *p* < 0.01) than warfarin users (1.16).

Table 1 summarizes the successful results of Kernel weighting in reducing bias for each comparison between single NOAC cohorts and patients prescribed warfarin. For each Kernel weighting, the mean and median standardized bias substantially decreased and the requested criteria for Rubin’s B and R were accomplished, with a negligible residual bias. Moreover, for each variable in any of the four comparisons between single NOAC and warfarin, the t-tests for equality of means in the two samples did not show any difference after Kernel weighting.

During the first two years after the index date, the crude mortality rate was 150.3 (95% CI 130.4–173.1) per 1000 person-years for warfarin users (*n* = 192 events), 114.6 (100.4–130.8) for edoxaban (*n* = 220), 111.1 (100.3–123.2) for apixaban (*n* = 364), 68.6 (57.4–82.0) for rivaroxaban (*n* = 121) and 68.1 (57.6–80.6) for dabigatran users (*n* = 136). The crude rate of hospitalizations for acute myocardial infarction was 93.0 per 1000 person-years for warfarin (77.2–112.0, *n* = 111 events), 68.4 (57.6–81.3) for dabigatran, 59.6 (43.6–65.9) for rivaroxaban, 58.6 (48.6–70.8) for edoxaban and 53.6 (43.6–65.9) for apixaban users.

There were 46 hospital admissions for stroke (crude rates 36.6, 27.4–48.8) among patients using warfarin, and crude rates were 27.2 (22.1–33.6), 25.4 (19.1–33.7) and 24.7 (18.3–33.3) for apixaban, edoxaban and rivaroxaban users, respectively.

Moreover, there were 60 hospitalizations for bleeding among warfarin users (crude rate 48.4, 37.6–62.3), 135 among those prescribed apixaban (42.3, 35.7–50.0), 73 for edoxaban (38.8, 30.9–48.8), 72 for dabigatran (36.9, 29.3–46.5) and 52 for rivaroxaban (30.1, 22.9–39.5).

Table 3 summarizes the results of the multivariate Cox models analyzing the risk (hazard ratio (HR)) of mortality and hospitalization for myocardial infarction, stroke and bleeding for each NOAC in comparison with warfarin. The risk of dying during the first two years of treatment, when adjusted for covariates, decreased from −25% to −49% (for rivaroxaban, HR = 0.51 (95%CI 0.41–0.62), for dabigatran 0.52 (0.42–0.64), for apixaban 0.69 (0.60–0.79) and for edoxaban 0.75 (0.63–0.89).

Regarding hospitalization for myocardial infarction during the first two years of follow-up, all NOACs showed adjusted HR lower than unity in comparison with warfarin (Table 3) (for edoxaban HR = 0.73 (0.57–0.94), for apixaban HR = 0.73 (0.60–0.88), for rivaroxaban HR = 0.74 (0.56–0.97) and for dabigatran HR = 0.84 (0.67–1.07)).

As for hospitalization for stroke, the short-term risk was reduced by 41% for dabigatran (HR = 0.59, 0.40–0.88) and by 26% for apixaban users (HR = 0.74, 0.56–0.98) in comparison with warfarin users. The risk was numerically but not significantly reduced for edoxaban (HR = 0.72, 0.50–1.05) and rivaroxaban (HR = 0.77, 0.51–1.15) users.

Finally, the risk of hospitalization for bleeding during the first two years of NOAC exposure was reduced for rivaroxaban users (HR = 0.67, 0.46–0.95) and numerically but not significantly lower than that for warfarin for the other NOACs users, from −22% to −19% (Table 3).

## 4. Discussion

In this large cohort of Italian NVAF patients based on the real-world population, we compared the short-term comparative performance of warfarin vs. four NOACs currently on the Italian market: dabigatran, rivaroxaban, apixaban and edoxaban. The purpose of the study was to provide evidence from clinical practice on both the effectiveness and the safety of NOACs compared to traditional warfarin. NOACs have shown efficacy and safety equal to or greater than warfarin in randomized clinical trials [26,27,28,29]. However, patients enrolled in those studies may be not representative of those in the general population [11]. Therefore, real-world observational studies are essential to verify the real performance of NOACs.

A recent meta-analysis, including 28 real-word studies, confirmed the experimental results for dabigatran, rivaroxaban and apixaban [30]. Notably, there was a large reduction of intracranial hemorrhages for all three NOACs, lower mortality for apixaban and dabigatran and fewer gastrointestinal and major bleeding for apixaban [30].

Edoxaban was authorized in the European Union in June 2015, and in Member States thereafter. In Italy, edoxaban entered into the market in late 2016, more than 2 years after dabigatran, 2 years after rivaroxaban and 18 months after apixaban [10]. Therefore, European real-world evidence for edoxaban was estimated and published more recently than for other NOACs [31,32,33,34]. Considering different times of availability for NOACs and consequently the variable number of users and lengths of follow-up, our analysis focused on short-term outcomes, limiting the comparison to new users and the first two years after the start of anticoagulant agents. This timeframe allowed for a fair comparison of NOACs, including edoxaban vs. warfarin.

We showed that all the four analyzed NOACs were more effective than warfarin in reducing two-year overall mortality, whereas apixaban, edoxaban and rivaroxaban also significantly decreased hospitalizations for myocardial infarction.

Additionally, dabigatran and apixaban were associated with a decreased risk of hospitalization for stroke. Furthermore, the hazard ratio of hospitalization for bleeding was below unity for all NOACs, although only rivaroxaban reached statistical significance [35].

Our data show that edoxaban was as effective as the other NOACs in significantly reducing mortality, as compared to warfarin. Moreover, edoxaban use was associated with numerically lower rates of hospitalizations for bleeding, confirming the results of a European observational study carried out on high-risk patients with NVAF routinely treated with edoxaban from 10 countries, that recently documented low rates of stroke, systemic embolism and major bleeding [36]. Furthermore, our results are in keeping with a recent meta-analysis based on patients with atrial fibrillation and valvular heart disease, also including one study on edoxaban that showed a decreased short-term risk of stroke and major bleeding for NOACs compared to warfarin [37].

The real-world picture of NOACs is changing rapidly, based on the availability of new drugs [13]. A recent survey conducted in Italy [38] using the Italian Medicines Agency monitoring registries showed that only 6.5% of the nearly 700,000 patients with NVAF treated with NOACs during 2012–2017 were treated with edoxaban, the least frequently used NOAC in comparison with rivaroxaban (33.8%), apixaban (31.1%) and dabigatran (28.6%). Our data, more updated and especially with a comparable timeframe and new user design, show a totally different picture, with apixaban (31.5%) and edoxaban (20.2%) being the most prescribed NOACs. The changing background makes it extremely important to continue the real-world evaluation of the effectiveness and safety of NOACs, providing up-to-date evidence on the population’s exposure to anticoagulants.

The present study has several limitations, which mainly relate to the observational nature of the data. Some unmeasured and residual confounding is likely to persist. However, this limitation is typical of all real-world studies. Moreover, we sought to address indication bias—for each NOAC or warfarin prescription—by a Kernel weighting, which adjusted each NOAC and warfarin patient based on a specific propensity score computed on the basis of many demographic and clinical covariates. We also included the number of comorbidities as a general measure of the health status. The check of bias changed before and after weighting measures based on the efficacy of such an approach.

Another limitation of this study, typical of all real-world studies based on administrative data, is represented by the exclusion of non-resident subjects who consume drugs, since it is not possible to identify the outcomes.

These exclusions are more theoretical than realistic (practically all patients in Italy are under the universal system, and the aim of this study was to analyze the patients registered in this administrative database).

As in all real-world studies, we do not have data on the quality of anticoagulation control among warfarin users, as reflected by time in the therapeutic range, which is important given its relationship with the efficacy and safety of VKA therapy. Moreover, the clinical outcomes of the study are based on specific hospitalization codes. Therefore, some errors cannot be excluded. Furthermore, the risks for clinical outcomes should also be considered, limited to those complications (including bleeding) so severe that they required hospitalization. Therefore, results may not be representative of the average real-world population. Moreover, no clues for the appropriateness of anticoagulant prescriptions may come from this study [1].

The prevalence of NVAF is strongly influenced by the age of patients [39]. As of 1 January 2019, the mean age of the Milan population was 45.3 years, and the percentage of citizens aged 65 years or over was 22.6%, and these values were very close to those of Italy as a whole, with 45.4 years and 22.6% respectively, according to the National Institute of Statistics (ISTAT; http://dati.istat.it, accessed on 30 July 2021). Furthermore, in the Framingham study, the risk of incident NVAF increased for BMI ≥ 30 kg/m^2^ [40]. The proportion of overweight and obese people in 2019 was 42.3% and 13.9% in Italy as a whole and 31.9% and 9.4% in Lombardy. These approximate demographic and clinical variables do not show the study population as very different from the Italian one.

Finally, the short-term follow-up, chosen for a fair comparison among the NOACs, did not allow for any prediction of late efficacy and safety.

## 5. Conclusions

Our results showed that in the real world, the NOACs are, in the first two years from starting treatment, more effective and safer than warfarin, at least for the analyzed outcomes. Their use is associated—in multivariate models—with a reduced risk of dying (for all NOACs), of hospitalization for myocardial infarction (apixaban, edoxaban and rivaroxaban), of stroke (dabigatran and apixaban) and of hospitalizations for bleeding (rivaroxaban).

## Figures and Tables

**Table 1:** Comparisons of each NOAC cohort with warfarin patients before and after Kernel weighting: mean and median bias, Rubin’s B and Rubin’s R.

	Dabigatran		Rivaroxaban		Apixaban		Edoxaban	
	Before	After	Before	After	Before	After	Before	After
Mean bias	10.8	1.2	11.7	1.9	13.2	2.1	11.4	1.3
Median bias	10.0	0.9	7.1	1.7	12.5	1.4	9.0	1.3
Rubin’s B (%)	48.5	5.6	44.4	5.9	45.8	8.3	51.7	5.4
Rubin’s R	0.84	1.08	1.03	1.16	1.15	1.24	1.05	1.13

**Table 2:** Characteristics of the atrial fibrillation patients for each analyzed anticoagulant agent.

	Warfarin	Dabigatran	Rivaroxaban	Apixaban	Edoxaban
*N*. (%)	1104 12.9	1636 19.2	1385 16.2	2693 31.5	1725 20.2
Mean age (SD)	75.4 11.3	75.4 10.9	73.9 * 12.1	79.0 ** 10.2	78.7 ** 10.3
% women	44.2	43.7	43.7	52.5 **	54.6 **
*N*. comorbidities Mean (SD)	2.3 1.1	2.0 0.9 **	2.0 1.0 **	2.0 1.0 **	2.1 1.0 **
CHA2DS2-VASc Mean(SD)	4.3 1.6	4.4 1.6	4.1 * 1.7	4.6 1.6	4.5 1.6
%PAI	3.7	5.0	3.6	4.3	3.9
%PPI	70.7	60.8 **	55.0 **	62.5 **	60.6 **
% in Milan	35.5	38.3	39.6 *	48.0 **	40.4 *
Follow up (years) (SD)	1.16 0.64	1.22 * 0.65	1.27 ** 0.64	1.22 * 0.67	1.11 0.65

For each NOAC, values are compared with those of warfarin with Student’s *t*-test or chi-square, when appropriate. PAI = platelet aggregation inhibitors excluding heparin, and PPI = proton pump inhibitors. Statistical significance of the comparisons: * *p* < 0.05; ** *p* < 0.001.

**Table 3:** Adjusted hazard ratios (HR ^) and 95% confidence intervals (95% CI) for overall mortality and hospitalization for myocardial infarction, stroke or bleeding for users of each analyzed anticoagulant agent vs. warfarin (reference).

	Warfarin	Dabigatran		Rivaroxaban		Apixaban		Edoxaban	
	Rate × 10^3^ (95% CI) HR	Rate × 10^3^ (95% CI)	HR (95% CI)	Rate × 10^3^ (95% CI)	HR (95% CI)	Rate × 10^3^ (95% CI)	HR (95% CI)	Rate × 10^3^ (95% CI)	HR (95% CI)
Mortality	150.3 (130.4–173.1)	68.1 (57.6–80.6)	0.52 (0.42–0.64)	68.6 (57.4–82.0)	0.51 (0.41–0.62)	111.1 (100.3–123.2)	0.69 (0.60–0.79)	114.6 (100.4–130.8)	0.75 (0.63–0.89)
MI	93.0 (77.2–112.0)	68.4 (57.6–81.3)	0.84 (0.67–1.07)	59.6 (43.6–65.9)	0.74 (0.56–0.97)	53.6 (43.6–65.9)	0.73 (0.60–0.88)	58.6 (48.6–70.8)	0.73 (0.57–0.94)
Stroke	36.6 (27.4–48.8)	20.1 (10.9–31.9)	0.59 (0.40–0.88)	24.7 (18.3–33.3)	0.77 (0.51–1.15)	27.2 (22.1–33.6)	0.74 (0.560.98)	25.4 (19.1–33.7)	0.72 (0.50–1.05)
Bleeding	48.4 (37.6–62.3)	36.9 (29.3–46.5	0.81 (0.59–1.11)	30.1 (22.9–39.5)	0.67 (0.46–0.95)	42.3 (35.7–50.0	0.80 (0.64–1.01)	38.8 (30.9–48.8	0.78 (0.57–1.05)

^ The models were weighted using the weights produced by the Kernel propensity score and included age, sex, comorbidities, use of platelets inhibitors, proton pump inhibitors and residence. MI = Myocardial Infarction.

## Data Availability

The data presented in this study are available on request from the corresponding author. The data are not publicly available due to they contain information that could compromise the privacy of research participants.
